# Clinical evolution and nutritional status in asthmatic children and adolescents enrolled in Primary Health Care

**DOI:** 10.1016/j.rpped.2015.02.005

**Published:** 2015

**Authors:** Rosinha Yoko Matsubayaci Morishita, Maria Wany Louzada Strufaldi, Rosana Fiorini Puccini

**Affiliations:** a Department of Pediatrics, Escola Paulista de Medicina, Universidade Federal de São Paulo (Unifesp), São Paulo, SP, Brazil

**Keywords:** Asthma, Children, Clinical evolution, Primary Health Care

## Abstract

**Objective:**

To evaluate the clinical evolution and the association between nutritional status and severity of asthma in children and adolescents enrolled in Primary Health Care.

**Methods:**

A retrospective cohort study of 219 asthmatic patients (3-17 years old) enrolled in Primary Care Services (PCSs) in Embu das Artes (SP), from 2007 to 2011. Secondary data: gender, age, diagnosis of asthma severity, other atopic diseases, family history of atopy, and body mass index. To evaluate the clinical outcome of asthma, data were collected on number of asthma exacerbations, number of emergency room consultations and doses of inhaled corticosteroids at follow-up visits in the 6th and 12th months. The statistical analysis included chi-square and Kappa agreement index, with 5% set as the significance level.

**Results:**

50.5% of patients started wheezing before the age of 2 years, 99.5% had allergic rhinitis and 65.2% had a positive family history of atopy. Regarding severity, intermittent asthma was more frequent (51.6%) and, in relation to nutritional status, 65.8% of patients had normal weight. There was no association between nutritional status and asthma severity (*p*=0.409). After 1 year of follow-up, 25.2% of patients showed reduction in exacerbations and emergency room consultations, and 16.2% reduced the amount of inhaled corticosteroids.

**Conclusions:**

The monitoring of asthmatic patients in Primary Care Services showed improvement in clinical outcome, with a decreased number of exacerbations, emergency room consultations and doses of inhaled corticosteroids. No association between nutritional status and asthma severity was observed in this study.

## Introduction

Asthma is a complex inflammatory disease, recognized as one of the most common chronic diseases of childhood. It is characterized by recurrent respiratory symptoms and it significantly impairs the quality of life. In Brazil, epidemiological studies carried out in recent decades indicate a trend of increasing prevalence of asthma in children and adolescents.^1^^-^ 5 In this same period, an increase in the prevalence of overweight and obesity in children and adolescents has also been observed in several countries and in Brazil, which constitutes a major public health problem.^6^ This analysis is supported by three national surveys that assessed nutritional status from 1974 to 2009, showing an increase in overweight and obesity in children and adolescents in all income groups and in all regions of Brazil.^7^ Obesity is a complex and multifactorial disease and it actively contributes to the development of cardiovascular diseases, arterial hypertension, diabetes mellitus and metabolic syndrome, in addition to exacerbating asthma.^8^^-^ 12

Epidemiological studies have suggested an association between obesity and asthma, more consistent in adults, revealing that obese asthmatic individuals have a higher frequency of exacerbation crises, emergency room consultations and need for higher doses of inhaled corticosteroids, as well as greater difficulty in controlling the disease.^11^^,^ 13 ^,^ 14 The association between obesity and asthma in children and adolescents is still controversial, and studies frequently use different methodologies, which explains, in part, the varying results obtained.^11^^,^ 15 ^-^ 21

In 1988, as part of the activities of the Teaching-Care Integration Program developed by Universidade Federal de São Paulo in the town of Embu das Artes, in the metropolitan region of São Paulo, the Care Program for Children with "Wheezing" was implemented in partnership with the Department of Health. The structure of this program considered as its principle the role of primary care in the management and monitoring of the most prevalent morbidities. The program's population consists of children and adolescents with recurrent wheezing, treated by a team of pediatricians, undergraduate medical students and medical residents in Pediatrics. The activities developed by the program include medical consultations, educational groups for family members and provision of medications for asthma control.

Ventura et al.^22^ evaluated this program in the 1988-1993 period and observed that before its implantation, most patients sought treatment for the disease only in periods of exacerbations and at emergency services. The authors verified that after 1 year of follow-up, children who maintained higher adherence to the program had benefited, despite the limited supply of asthma medications available in the public health system in that period, which was restricted to the distribution of bronchodilators, theophylline and systemic corticosteroids only. This situation has changed since 2009, with *Portaria* N° 2981 of the Ministry of Health, which provides for the regular provision of medications for asthma control during crises and for continuous use by the municipality.^23^

Considering the nutritional transition in Brazil, with the reduction in the prevalence of malnutrition and increase in the prevalence of obesity,^7^ a phenomenon which was also observed in the municipality of Embu das Artes,^24^ and considering a possible association between obesity and worsening of asthma, with more exacerbation crises and visits to the emergency room in pediatric patients,^15^^-^ 21 the present study aimed to evaluate the clinical evolution and the association between nutritional status and disease severity in children and adolescents with asthma enrolled at and followed in a Primary Care Service (PCS) of the Brazilian Public Health System (SUS).

## Method

This retrospective cohort involved children aged 3-17 years, enrolled in the Care Program for Children with "Wheezing" from 1 January 2007 to 31 December 2011, in two PCSs in the municipality of Embu das Artes. Secondary data obtained from the first consultation were collected from patients’ records: age, gender, age at first wheezing crisis and other atopic diseases (rhinitis, dermatitis), previous hospitalizations for respiratory diseases, birth weight, family history of atopy (asthma, rhinitis and dermatitis), and diagnosis of asthma severity using the clinical criteria of the IV Brazilian Guidelines for Asthma Management, 2006 (intermittent asthma, mild persistent, moderate persistent and severe persistent asthma).^25^ Regarding the clinical course of the disease, the number of emergency room consultations and asthma attacks were collected, in addition to inhaled corticosteroid doses according to the 2012 GINA Consensus equipotential^2^ in the 6th and 12th months of follow-up. The levels of asthma control were classified in the 12th month of follow-up according to the 2012 GINA Consensus.^2^

Anthropometric data, such as weight (kg), height (cm) and the calculation of body mass index (BMI) (kg/m^2^) *z*-score based on the WHO curves of 2006 and 2007, using the Anthro and Anthro Plus programs,^26^^,^ 27 were obtained from the medical records of the 1st consultation. The cutoff points used were according to *z*-score: ≥−3 to <−2: thinness; ≥−2 to <+1: normal weight; ≥+1 to <+2: risk of overweight (<5 years) and overweight (≥5 years); ≥+2 to <+3: overweight (<5 years) and obesity (≥5 years); ≥+3: obesity (<5 years) and severe obesity (≥5 years).^26^^,^ 27

Patients were considered as cases of loss of follow-up after failing to come to consultation for more than 6 months since the last visit and discharge was defined for cases of asthma controlled without the use of inhaled corticosteroids.

Regarding the study procedure, a total of 439 children were identified with a first scheduled consultation in the period; after 92 patients were excluded as they were younger than 3 years and/or had other pulmonary diseases and syndromes, 347 patients who met the inclusion criteria remained. Of these, there was a loss of 95 individuals who missed the first consultation, 32 records were not found, and there was one death, thus totalling 219 patients selected for the study and who attended the 1st consultation.

After 1 year of follow-up, 39 patients (17.8%) abandoned treatment. Four patients (1.8%) with intermittent asthma were discharged from the program during this period; one patient with intermittent asthma moved to another city. During this period, 88 patients (40%) missed at least one consultation and thus, 131 patients came to all proposed consultations, having the complete data to be used in the analysis of the clinical course of the disease.

For the statistical analyses, chi-square tests were used to compare categorical variables, with the level of significance being set at 5% and two-tailed statistical tests. The Kappa index was used to assess the number of crises, emergency room consultations and doses of medications used between the first and second semester of the program follow-up, with lack of agreement being considered when the value was zero (*κ*=0) and perfect agreement with Kappa index =1. The standard error of the Kappa index of 5% allowed the estimation of the statistical significance and the 95% confidence interval.

This study was approved by the Institutional Review Board of the Department of Health of Embu das Artes and was also approved by the Institutional Review Board of UNIFESP, n. 1821/11.

## Results

The 219 patients who attended the 1st consultation had a mean age of 6.65 years ±4.89 SD (3-17 years), with most being male (58.4%); 9.3% had low birth weight and 50.5% of patients started wheezing before 2 years of age; 91.7% had other atopic conditions, almost all of them with allergic rhinitis (99.5%), and 46% reported previous hospitalization at the start of follow-up. Family history of atopy was positive in 65.2% of patients. As for disease severity, intermittent asthma was the most frequent type (51.6%), and in relation to nutritional status, normal weight predominated (65.8%) among patients. The calculated BMI had a mean ± SD of 17.22 ± 3.34 (11.98-36.22 kg/m^2^), and *z*-score of 0.48 ± 5.20 (−2.72 to 5.20).

Patient distribution according to the nutritional status and the severity of asthma in the 1st consultation is described in Table 1. It should be noted that severe asthma was diagnosed only in patients with overweight and obesity. After statistical analysis, it was observed that there was no significant association between nutritional status and asthma severity (*p*=0.409).

**Table 1:** Nutritional status and asthma severity in the 1st consultation at the Care Program for Children with “Wheezing”, Embu das Artes.

Nutritional status	Asthma severity					
	Exercise	Intermittent	Mild	Moderate	Severe	
	*n*=3 (%)	*n*=113 (%)	*n*=54 (%)	*n*=46 (%)	*n*=3	%
Thinness	0 (0.0)	1 (20.0)	2 (40.0)	2 (40.0)	0	0.0
Normal weight	2 (1.4)	81 (56.3)	34 (23.6)	27 (18.8)	0	0.0
Overweight risk	0 (0.0)	8 (40.0)	6 (30.0)	6 (30.0)	0	0.0
Overweight	1 (2.9)	16 (47.1)	8 (23.5)	7 (20.6)	2	5.9
Obesity	0 (0.0)	7 (43.8)	4 (25.0)	4 (25.0)	1	6.2

*p*-value=0.409.

In relation to asthma attacks, approximately 20% of patients had no exacerbations in the 1st and 2nd semesters. There was no agreement between the two moments due to the known heterogeneous disease progression (*κ*=0.060 and *p*=0.375); however, 25.2% of the patients improved exacerbations, and 19.1% started having more crises in the 2nd semester (Table 2). It is noteworthy that these patients started the program follow-up in different months of the year, thus minimizing the influence of seasonality on the frequency of exacerbations.

**Table 2:** Number of asthma crises in the 1st and 2nd semesters of follow-up of patients enrolled in the Care Program for Children with “Wheezing”, Embu das Artes.

			Number of crises – 2nd semester			Total
			None	1–5	≥6 crises	
**Number of crises – 1^st^ semester**	*None*	*n*	9	16	1	26
		%	6.9	12.2	0.8	19.8
	*1–5*	*n*	17	63	8	88
		%	13.0	48.1	6.1	67.2
	*≥6 crises*	*n*	2	14	1	17
		%	1.5	10.7	0.8	13.0
*Total*		*n*	28	93	10	131
		%	21.4	71.0	7.6	100.0

*κ*=0.060; *p*-value=0.375.

Table 3 shows the number of emergency room consultations due to asthma attacks per patient between the 1st and 2nd semesters. The Kappa index showed an agreement between the two periods (*p*=0.013), but with a value of *κ*=0.180, i.e., a poor agreement. When analyzing all the answers, 57.3% of patients had the same number of emergency room consultations in both moments; 25.2% showed improvement and 17.6% showed worsening of the clinical picture, with more emergency room consultations, with this fact explaining the lack of agreement between the results.

**Table 3:** Number of patients’ visits to the emergency department (ED) in the 1st and 2nd semesters of follow-up at the Care Program for Children with “Wheezing”, Embu das Artes.

			Number of visits to the ED – 2nd semester			Total
			None	1–2 visits	3 or more	
**Number of visits to ED – 1st semester**	*None*	*n*	57	16	3	76
		%	43.5	12.2	2.3	58.0
	*1–2 visits*	*n*	24	17	4	45
		%	18.3	13.0	3.1	34.4
	*3 or more*	*n*	3	6	1	10
		%	2.3	4.6	0.8	7.6
*Total*		*n*	84	39	8	131
		%	64.1	29.8	6.1	100.0

*κ*=0.180; *p*-value=0.013.

Table 4 describes the doses of inhaled corticosteroids used by asthma patients in the 1st and 2nd semesters. The observed results showed that there was an agreement between the two moments in time (*p*=0.001), with a value of *κ*=0.569, indicating moderate agreement. It can be observed that 73.8% of patients remained with the same dose of corticosteroids in the 1st and 2nd semesters, indicating a high correlation between the results in the two moments; 10.0% of patients showed worsening and it was necessary to start using inhaled corticosteroids in the 2nd semester, with 2.3% using medium and 4.6%, high doses. Of the 16.2% of patients who improved, 8.4% reduced the amount and 7.7% stopped using medication in the 2nd semester of follow-up. A patient using the association of inhaled corticosteroids/long-acting bronchodilator in the 2nd semester was excluded from this table to allow statistical analysis. Additionally, it was observed that 11.3% of patients in the 1st semester showed irregular use of inhaled corticosteroids, and 8% in the 2nd semester of follow-up.

**Table 4:** Inhaled corticosteroid doses used by patients in the 1st and 2nd semester followed at the Care Program for Children with “Wheezing”, Embu das Artes.

			Used inhaled corticosteroids – 2nd semester				Total
			Did not use	Low dose	Medium dose	High dose	
Used inhaled corticosteroids – 1st semester	*Did not use*	*n*	58	1	3	6	68
		%	44.6	0.8	2.3	4.6	52.3
	*Low dose*	*n*	1	1	0	0	2
		%	0.8	0.8	0.0	0.0	1.5
	*Medium dose*	*n*	4	2	7	3	16
		%	3.1	1.5	5.4	2.3	12.3
	*High dose*	*n*	5	0	9	30	44
		%	3.8	0.0	6.9	23.1	33.8
*Total*		*n*	68	4	19	39	130
		%	52.3	3.1	14.6	30.0	100.0

*κ*=0.569; *p*-value<0.001.

Table 5 shows the levels of asthma control and nutritional status at 12 months of follow-up of 131 patients. Obese patients showed more controlled asthma (53.3%), followed by partially controlled (40.0%) and uncontrolled (6.7%). According to the analysis, it was observed that there was no significant association between nutritional status and asthma control levels (*p*=0.057).

**Table 5:** Nutritional status and disease control levels in the 2nd semester of follow-up.

		Asthma control								*p*-value
		Controlled			Partially controlled			Uncontrolled		
		*n*	%		*n*	%		*n*	%	
*Nutritional status*	Thinness	1	100.0		0	0.0		0	0.0	0.057
	Normal weight	44	57.9		22	28.9		10	13.2	
	Overweight risk	5	50.0		2	20.0		3	30.0	
	Overweight	18	62.1		8	27.6		3	10.3	
	Obesity	8	53.3		6	40.0		1	6.7	

## Discussion

Given the increasing prevalence of asthma^2^^-^ 5 and of obesity^6^^,^ 7 in childhood, this study sought to establish an association between the two, the impact on disease evolution and the effects of therapeutic intervention after a 1-year follow-up of patients enrolled in the Care Program for Children with "Wheezing".

When analyzing the nutritional status of asthmatic patients in this study, it is observed that the results contrast with the findings of Ventura et al.^28^ in the same program of the municipality, although the nutritional status classification criteria are different in the two assessed moments, including regarding the use of the term "malnutrition" and not thinness, as currently recommended by the WHO^26^^,^ 27; the authors did not report any patients with excess weight, and 38% were malnourished according to that assessment, which reinforces the observation of the nutritional transition among the patients in this program, as reported in the national Household Budget Survey of 2008-2009.^7^

In this study, when assessing the nutritional status and asthma severity in 1st consultation, the severe form of the disease was observed only in patients with excess weight, while Ventura et al. observed the highest prevalence of malnutrition in the moderate/severe asthma group in the 1988-1993 period in the same municipality, despite the differences in nutritional classification criteria used at the time.^28^

Regarding the clinical diagnosis of disease severity in the 1st consultation, the highest frequency was of intermittent asthma (51.5%), while severe asthma was observed in only 1.4%, a similar rate to that reported in the IV Brazilian Guidelines for Asthma Management, 2006.^25^Simões et al.,^29^ in the municipality of Salvador, state of Bahia, described a higher prevalence of mild (40%) and severe (10.8%) asthma and lower prevalence of intermittent (36%) and moderate (12.8%) asthma.

In the present study, we assessed the influence of high BMI and asthma severity, as well as disease control after a year of follow-up of patients enrolled in the program. The analyses showed no associations between nutritional status and asthma severity, as well as between nutritional status and disease control.

Studies on the association between asthma and obesity show heterogeneous results due to the use of different methods for asthma severity classification, with some reporting symptoms of wheezing, while others use medical diagnosis or objective measurements such as spirometry, therefore showing varied results. Farah and Salome,^11^ in a review article, pointed out the controversies on the association between obesity and worse asthma severity. Cassol et al.^16^ carried out a population-based study with adolescents in a city in southern Brazil and did not find an association between increased BMI and asthma symptom worsening. In the USA, Ross et al.^17^ did not find any differences in pulmonary function between obese and nonobese children and adolescents with asthma. On the other hand, other studies have shown an association between high BMI and higher asthma severity.^18^^,^ 19

Regarding asthma control, the association between obesity and worse disease control is not well established yet. Quinto et al.^21^observed that children and adolescents with overweight and obesity had worse asthma control, with more exacerbations and more frequent use of rescue medications. Kattan et al.^20^ described an association between worse asthma control and obesity in the female gender in a study of adolescents with asthma. Other authors found no differences in levels of asthma control between obese and nonobese patients.^17^^,^ 30

The present study assessed the evolution of asthma considering patients who attended all proposed consultations, considering that the characteristics of these patients may be different from those who left the program and the ones who missed the consultations, which may have limited the analyses of disease evolution. The quantification of the number of crises may have been influenced by recall bias, especially when exacerbations occurred several times during one semester; the emergency room consultations are important events in a patient's life, making records more reliable. On the other hand, patients started the Program follow-up in different months of the year, thus reducing the bias of asthma exacerbations due to seasonality.

During 1 year of follow-up, 25.2% of patients reduced the number of crises and emergency room consultations. Of the patients who showed worsening, only 19.1% had more crises, and 17.6% required more emergency room consultations in that period. It was observed that the proportion of asthmatics who showed improvement was higher than the ones who showed worsening, demonstrating the benefits of monitoring in those patients who joined the program.

Ventura et al.^22^ verified that patients with moderate and severe asthma had a smaller number of crises, when analyzing the period before and after 1 year of follow-up, in the same PCSs, from 1988 to 1993. These authors also observed that time of follow-up influenced the evolution of moderate asthma, showing clinical improvement with time longer than 1 year. Moreover, the time of follow-up shorter or longer than 1 year showed no difference in the evolution of severe asthmatics.^22^ The observed results are in agreement with the retrospective cohort study carried out by Fontes et al.,^31^ who evaluated the follow-up of asthmatic children and adolescents in a Pulmonology Service in the city of Belo Horizonte, State of Minas Gerais, observing a significant reduction in the frequency of hospitalizations and emergency room visits for asthma during a 1-year period.

According to this study, most patients showed treatment adherence. An important part of these results should be attributed to the provision of asthma medications by the Public Health System PCSs, including the municipality of Embu das Artes, since 2009, with the *Portaria* N° 2981 of the Ministry of Health.^23^ Progress has been made in Public Health Policies with health managers’ acknowledgment that asthma is one of the most common chronic diseases, by making available inhaled medications such as corticosteroids with or without a combination with long-acting bronchodilators, rescue medications such as short-acting β-agonists and oral corticoids to patients with asthma.

Improved adherence to the program by patients and family members was demonstrated by the decrease in the abandonment rate, from 53.2%^22^ since its implementation in both PCSs to currently 17.8%. This finding was also described by Dalcin et al. in asthmatic patients recruited from an outpatient clinic of a hospital in the city of Porto Alegre, state of Rio Grande do Sul, also observing that access to medication and adequate use of inhaled corticosteroids were associated with asthma control.^30^

However, despite the consistent improvement in program adherence and follow-up, this study has some limitations, such as obtaining some secondary data (medical records), and the loss of information on patients that missed appointments or abandoned the program, while study data was being collected. In spite of these limitations, we can conclude that the study showed no associations between nutritional status and asthma severity and control. Adherence to treatment resulted in a decreased number of asthma crises, emergency room consultations during the follow-up period, in addition to decreasing the doses of inhaled corticosteroids. This study emphasizes the importance and feasibility of asthmatic patients follow-up in PCSs. Moreover, the implementation of public policies that facilitated the introduction of specific medications for asthma treatment, the most common chronic disease in childhood, in Primary Health Care contributed to the favorable outcome of this evolution.
