# Supplementary material for: Clinical evolution and nutritional status in asthmatic children and adolescents enrolled in Primary Health Care
Source: Rev Paul Pediatr. 2015 Oct-Dec;33(4):387–93. doi: 10.1016/j.rpped.2015.02.005 (PMC4685557; doi:10.1016/j.rpped.2015.02.005)
Supplement: Translation

# Translation: Evolução clínica e estado nutricional de crianças e adolescentes asmáticos acompanhados em Unidade Básica de Saúde

**Authors:** Rosinha Yoko Matsubayaci Morishita, Maria Wany Louzada Strufaldi, Rosana Fiorini Puccini

**Affiliations:** a Departamento de Pediatria, Escola Paulista de Medicina, Universidade Federal de São Paulo (Unifesp), São Paulo, SP, Brasil

**Keywords:** Asma, Crianças, Evolução clínica, Atenção básica à saúde

## Abstract

**Objetivo:**

Avaliar a evolução clínica e a associação entre o estado nutricional e a gravidade da asma em crianças e adolescentes matriculados em Unidades Básicas de Saúde (UBS).

**Métodos:**

Estudo de coorte retrospectiva com 219 pacientes asmáticos (3-17 anos), matriculados em UBS do município de Embu das Artes (SP), de 2007 a 2011. Dados secundários: sexo, idade, diagnóstico de gravidade da asma, outras atopias, antecedentes familiares de atopia, índice de massa corporal. Para avaliar a evolução da asma foram coletados número de crises de asma, número de atendimentos de urgência e doses de corticoide inalatório no 6° e 12° mês de acompanhamento. A análise estatística incluiu testes de qui-quadrado e índice de concordância Kappa, com nível de significância de 5%.

**Resultados:**

Dos pacientes, 50,5% iniciaram a sibilância antes dos dois anos; 99,5% apresentaram rinite alérgica e 65,2% antecedente familiar para atopia positivo. Quanto à gravidade, a asma intermitente foi mais frequente (51,6%); em relação ao estado nutricional, 65,8% dos pacientes eram eutróficos. Não houve associação entre o estado nutricional e a gravidade da asma (*p*=0,409). Após um ano de acompanhamento, 25,2% dos pacientes reduziram as exacerbações e os atendimentos nas urgências e 16,2% reduziram a quantidade de corticoide inalatório.

**Conclusões:**

O acompanhamento dos pacientes asmáticos em UBS demonstrou melhoria da evolução com redução do número de exacerbações, dos atendimentos nas urgências e das doses de corticoide inalatório. Não houve associação entre o estado nutricional e gravidade da asma.

## Introdução

A asma é uma doença inflamatória complexa, reconhecida como uma das doenças crônicas mais comuns da infância. Caracteriza-se por manifestações respiratórias recorrentes e compromete de forma significativa a qualidade de vida. No Brasil, estudos epidemiológicos feitos nas últimas décadas apontam para uma tendência de incremento da prevalência de asma em crianças e adolescentes.^1^^-^ 5 Nesse mesmo período, tem sido observado também um aumento na prevalência de sobrepeso e obesidade em crianças e adolescentes em diversos países do mundo e no Brasil, o que se constitui em importante problema de saúde pública.^6^ Essa condição é realçada a partir de três inquéritos nacionais de avaliação do estado nutricional, de 1974 a 2009, que demonstraram elevação de sobrepeso e obesidade em crianças e adolescentes, em todos os grupos de renda e em todas as regiões brasileiras.^7^ A obesidade é uma doença multifatorial e complexa e contribui ativamente para o desenvolvimento de doenças cardiovasculares, hipertensão arterial, *diabetes melittus* e síndrome metabólica, além de exacerbar a asma.^8^^-^ 12

Estudos epidemiológicos sugerem uma associação entre a obesidade e a asma, mais consistente em adultos, e observam que os obesos asmáticos apresentam maior frequência de crises de exacerbações, atendimentos nas urgências e necessidade de maiores doses de corticoides inalatórios, além de maior dificuldade no controle da doença.^11^^,^ 13 ^,^ 14 A associação entre obesidade e asma em crianças e adolescentes ainda é controversa e os estudos, muitas vezes, usam metodologias diferentes, o que explica, em parte, a obtenção de resultados variados.^11^^,^ 15 ^-^ 21

Em 1988, como parte das atividades do Programa de Integração Docente-Assistencial desenvolvido pela Universidade Federal de São Paulo (Unifesp) no município Embu das Artes, na Região Metropolitana de São Paulo, foi implantado, em parceria com a Secretaria Municipal de Saúde, o Programa de Atenção às Crianças Portadoras de "Chiado no Peito". A estruturação desse programa considerou como princípio o papel da atenção básica na assistência e no acompanhamento das morbidades mais prevalentes. A população do Programa é constituída por crianças e adolescentes com sibilâncias recorrentes, que são assistidas por uma equipe composta por pediatras, alunos da graduação do curso de medicina e médicos residentes de pediatria. As atividades desenvolvidas pelo Programa incluem consultas médicas, grupos educativos para os familiares e fornecimento das medicações para o controle da asma.

Ventura et al.^22^ avaliaram esse Programa de 1988 a 1993 e observaram que antes da implantação a maioria dos pacientes buscava o tratamento da doença apenas nos períodos de exacerbações e em serviços de urgência. Os autores verificaram que após um ano de acompanhamento as crianças que mantiveram maior aderência foram beneficiadas, apesar da limitação de medicamentos disponíveis na rede pública de saúde naquele período, com distribuição apenas de broncodilatadores, teofilinas e corticoides sistêmicos. Essa situação se modificou a partir de 2009, com a Portaria n° 2.981 do Ministério da Saúde, que prevê a disponibilização com regularidade de medicamentos para controle da asma nas crises e para uso contínuo pelo município.^23^

Considerando a transição nutricional no Brasil, com redução da prevalência da desnutrição e o aumento da obesidade,^7^fenômeno observado, também, no município de Embu das Artes,^24^ e tendo em vista uma possível associação entre a obesidade e a asma com piora da evolução, com mais crises de exacerbações e atendimentos de urgência na faixa pediátrica,^15^^-^ 21 o presente estudo teve como objetivo avaliar a evolução clínica e a associação do estado nutricional com a gravidade da doença em crianças e adolescentes asmáticos matriculados e acompanhados em Unidade Básica de Saúde (UBS).

## Método

O presente estudo, de coorte retrospectiva, envolveu o universo de crianças maiores de três anos até adolescentes de 17 anos, matriculados no Programa de Atenção às Crianças Portadoras de "Chiado no Peito", de 1 de janeiro de 2007 a 31 de dezembro de 2011, em duas UBS, no município de Embu das Artes. Dos prontuários dos pacientes foram coletados dados secundários da primeira consulta: idade, sexo, idade na primeira crise de sibilância e outras doenças atópicas (rinite, dermatite), internações prévias por doenças respiratórias, peso ao nascer, antecedente familiar de atopia (asma, rinite e dermatite) e diagnóstico da gravidade da asma com o uso dos critérios clínicos da IV Diretrizes Brasileiras para o Manejo da Asma, 2006 (asma intermitente, persistente leve, persistente moderada e persistente grave).^25^ Em relação à evolução clínica da doença, foram coletados os números de atendimentos nos serviços de urgência e as crises de asma, além das doses de corticoide inalatório segundo as equipotências do Consenso GINA 2012^2^ no sexto e no 12° mês de acompanhamento. Os níveis de controle da asma foram classificados no 12° mês de seguimento de acordo com o Consenso GINA 2012.^2^

Os dados de antropometria, como o peso (kg), a estatura (cm) e o cálculo de Índice de Massa Corporal (IMC) (kg/m^2^) em escore z segundo as curvas de OMS 2006 e 2007, com o uso dos programas Anthro e Anthro Plus,^26^^,^ 27 foram obtidos dos registros dos prontuários na primeira consulta. Os pontos de corte usados foram de acordo com z-escore: ≥−3 a <−2: magreza; ≥−2 a <+1: eutrofia; ≥+1 a <+2: risco de sobrepeso (<5 anos) e sobrepeso (≥5 anos); ≥+2 a <+3: sobrepeso (<5 anos) e obesidade (≥5 anos); ≥+3: obesidade (<5 anos) e obesidade grave (≥5 anos).^26^^,^ 27

Foi considerado abandono do Programa o não comparecimento por mais de seis meses da última consulta e a alta foi definida para os casos de asma controlada sem necessidade de uso de corticoide inalatório.

Em relação ao procedimento do estudo, foram identificadas 439 crianças com primeira consulta agendada no período, excluídos 92 pacientes menores de três anos e/ou com outras doenças pulmonares e síndromes. No total, 347 pacientes preencheram os critérios de inclusão. Desses pacientes, houve perda de 95 indivíduos que faltaram à primeira consulta, 32 prontuários não localizados e um óbito. Foram então selecionados para o estudo 219 pacientes que compareceram à primeira consulta.

Após um ano de acompanhamento, 39 pacientes (17,8%) abandonaram o tratamento. Quatro (1,8%) com asma intermitente receberam alta do Programa nesse período; um paciente com asma intermitente mudou para outro município. Durante esse período, 88 pacientes (40%) faltaram pelo menos a uma consulta e, assim, 131 compareceram a todas as consultas propostas e apresentaram os dados completos para analisar a evolução clínica da doença.

Para as análises estatísticas foram usados testes de qui-quadrado para comparar as variáveis categóricas, o nível de significância foi de 5% e o teste estatístico bicaudal. O índice de concordância Kappa foi usado para avaliar os números de crises, atendimentos de urgência e as doses de medicamentos usadas entre o primeiro e segundo semestre de acompanhamento no Programa. Foi considerada não concordância quando o valor calculado foi igual a zero (κ=0) e concordância perfeita com índice de Kappa igual um. O erro padrão do índice de Kappa de 5% permitiu estimar a significância estatística e o intervalo de confiança de 95%.

Este estudo foi aprovado pelo Comitê de Ética da Secretaria Municipal de Saúde de Embu das Artes e também aprovado pelo Comitê de Ética em Pesquisa da Unifesp, n° 1.821/11.

## Resultados

Os 219 pacientes que compareceram à primeira consulta apresentaram média de 6,65 anos ±4,89 DP (3-17 anos); a maioria era do sexo masculino (58,4%); 9,3% nasceram com baixo peso e 50,5% iniciaram a sibilância antes dos dois anos; 91,7% apresentaram outras atopias, quase todos com rinite alérgica (99,5%), e 46% relataram internação prévia ao início do acompanhamento. A história familiar para atopia foi positiva em 65,2% dos pacientes. Quanto à gravidade da doença, a asma intermitente foi mais frequente (51,6%) e em relação à condição nutricional houve predominância de eutrofia (65,8%). Entre os pacientes o IMC calculado teve média ± DP de 17,22±3,34 (11,98-36,22Kg/m^2^) e escore z de 0,48±5,20 (−2,72 a 5,20).

A distribuição dos pacientes segundo o estado nutricional e a gravidade da asma na primeira consulta está descrita na Tabela 1. Ressalta-se que a asma grave foi diagnosticada somente entre os pacientes com sobrepeso e obesidade. Após as análises estatísticas verificou-se que não houve associação significante entre o estado nutricional e a gravidade da asma (*p*=0,409).

**Tabela 1:** Estado nutricional e gravidade da asma na primeira consulta do Programa de Atenção às Crianças Portadoras de “Chiado no Peito”, Embu das Artes

	Gravidade da asma													
	Exercício			Intermitente			Leve			Moderada			Grave	
Estado nutricional	N=3	%		N=113	%		N=54	%		N=46	%		N=3	%
Magreza	0	0,0		1	20,0		2	40,0		2	40,0		0	0,0
Eutrofia	2	1,4		81	56,3		34	23,6		27	18,8		0	0,0
Risco de sobrepeso	0	0,0		8	40,0		6	30,0		6	30,0		0	0,0
Sobrepeso	1	2,9		16	47,1		8	23,5		7	20,6		2	5,9
0besidade	0	0,0		7	43,8		4	25,0		4	25,0		1	6,2

*p*‐valor=0,409.

Em relação às crises de asma, cerca de 20% dos pacientes não apresentaram exacerbações no primeiro e no segundo semestre. Não houve concordância entre os dois momentos pela evolução sabidamente heterogênea da doença (κ=0,060 e *p*=0,375), porém 25,2% dos pacientes melhoraram as exacerbações e 19,1% passaram a ter mais crises no segundo semestre (Tabela 2). Ressalta-se que esses pacientes iniciaram o seguimento no Programa em meses diferentes do ano e minimizaram, assim, a influência da sazonalidade na frequência das exacerbações.

**Tabela 2:** Número de crises de asma no primeiro e no segundo semestre de acompanhamento dos pacientes matriculados no Programa de Atenção às Crianças Portadoras de “Chiado no Peito”, Embu das Artes

			Número de crises – 2̊ semestre			Total
			Nenhuma	1 a 5	≥6 crises	
Número de crises ‐ 1̊ semestre	Nenhuma	n	9	16	1	26
		%	6,9	12,2	0,8	19,8
	1 a 5	n	17	63	8	88
		%	13,0	48,1	6,1	67,2
	≥6 crises	n	2	14	1	17
		%	1,5	10,7	0,8	13,0
Total		n	28	93	10	131
		%	21,4	71,0	7,6	100,0

Kappa=0,060; *p*‐valor=0,375.

A Tabela 3 apresenta o número de atendimentos em serviços de urgência decorrente de crises de asma por paciente entre o primeiro e o segundo semestre. O índice de concordância Kappa demonstrou uma concordância entre os dois períodos (*p*=0,013), porém com um valor de κ=0,180; uma concordância pobre. Ao analisar todas as respostas, 57,3% dos pacientes tiveram número de idas aos serviços de urgência igual nos dois momentos; 25,2% apresentaram melhoria e 17,6% pioraram o quadro, com mais atendimentos nas urgências. Essa é a explicação para a não concordância entre os resultados.

**Tabela 3:** Número de idas ao serviço de urgência (SU) pelos pacientes no primeiro e segundo semestre de acompanhamento no Programa de Atenção às Crianças Portadoras de “Chiado no Peito”, Embu das Artes

			Número de idas ao SU – 2̊ semestre			Total
			Nenhuma	1 a 2 idas	3 ou mais	
Número de idas ao SU – 1̊ semestre	Nenhuma	n	57	16	3	76
		%	43,5	12,2	2,3	58,0
	1 a 2 idas	n	24	17	4	45
		%	18,3	13,0	3,1	34,4
	3 ou mais	n	3	6	1	10
		%	2,3	4,6	0,8	7,6
Total		n	84	39	8	131
		%	64,1	29,8	6,1	100,0

Kappa=0,180; *p*‐valor=0,013.

A Tabela 4 descreve as doses de corticoide inalatório usadas pelos pacientes asmáticos no primeiro e no segundo semestre. Pelos resultados observados pode-se verificar que houve concordância entre os dois momentos (*p*=0,001), com um valor de κ=0,569, o que aponta concordância moderada. Nota-se que 73,8% dos pacientes permaneceram com a mesma dose de corticoide no primeiro e no segundo semestre, o que aponta alta concordância entre os resultados nos dois momentos; 10% dos pacientes pioraram e houve a necessidade de iniciar uso do corticoide inalatório no segundo semestre; 2,3% usaram dose média e 4,6% dose alta. Dos 16,2% pacientes que melhoraram, 8,4% reduziram a quantidade e 7,7% pararam de usar medicação no segundo semestre de acompanhamento. Um paciente que usou associação corticoide inalatório/broncodilatador de longa duração no segundo semestre foi excluído dessa tabela para possibilitar a análise estatística. Além disso, foi observado que 11,3% dos pacientes no primeiro semestre apresentaram uso irregular de corticoide inalatório e 8% no segundo semestre de seguimento.

**Tabela 4:** Doses de corticoide inalatório usadas pelos pacientes no primeiro e segundo semestre acompanhados no Programa de Atenção às Crianças Portadoras de “Chiado no Peito”, Embu das Artes

			Usou corticoide inalatório – 2̊ semestre				Total
			Não usou	Dose baixa	Dose média	Dose alta	
Usou corticoide inalatório – 1̊ semestre	Não usou	n	58	1	3	6	68
		%	44,6	0,8	2,3	4,6	52,3
	Dose baixa	n	1	1	0	0	2
		%	0,8	0,8	0,0	0,0	1,5
	Dose média	n	4	2	7	3	16
		%	3,1	1,5	5,4	2,3	12,3
	Dose alta	n	5	0	9	30	44
		%	3,8	0,0	6,9	23,1	33,8
Total		n	68	4	19	39	130
		%	52,3	3,1	14,6	30,0	100,0

Kappa=0,569; *p*‐valor<0,001.

A Tabela 5 apresenta os níveis de controle da asma e o estado nutricional no 12° mês de acompanhamento dos 131 pacientes. Os obesos apresentaram maior proporção de asma controlada (53,3%), seguida de parcialmente controlada (40%) e a forma não controlada (6,7%). Feitas as análises, demonstrou-se que não houve associação significante entre estado nutricional e níveis de controle da asma (*p*=0,057).

**Tabela 5:** Estado nutricional e os níveis de controle da doença no segundo semestre do acompanhamento

		Controle de asma								*p*‐valor
		Controlada			Parcialmente controlada			Não controlada		
		n	%		n	%		n	%	
Estado nutricional	Magreza	1	100,0		0	0,0		0	0,0	0,057
	Eutrofia	44	57,9		22	28,9		10	13,2	
	Risco de sobrepeso	5	50,0		2	20,0		3	30,0	
	Sobrepeso	18	62,1		8	27,6		3	10,3	
	Obesidade	8	53,3		6	40,0		1	6,7	

## Discussão

Diante da crescente prevalência de asma^2^^-^ 5 e de obesidade^6^^,^ 7 durante a infância, este estudo buscou estabelecer as relações entre ambas, o impacto sobre a evolução da doença e os efeitos da intervenção terapêutica após um ano de acompanhamento dos pacientes matriculados no Programa Atenção às Crianças Portadoras de "Chiado no Peito".

Ao analisar a condição nutricional dos pacientes asmáticos deste estudo, nota-se que os resultados contrastam com os achados de Ventura et al.^28^ no mesmo Programa do município, embora os critérios de classificação da condição nutricional sejam diferentes nos dois momentos estudados, até quanto ao uso do termo desnutrição, e não magreza, como atualmente é recomendado pela OMS;^26^^,^ 27 os autores não observaram paciente com excesso de peso e 38% eram desnutridos naquela avaliação, o que reforça a observação do fenômeno da transição nutricional entre os pacientes desse Programa, como relatado em inquérito nacional, a Pesquisa de Orçamentos Familiares 2008-2009.^7^

No presente estudo, ao avaliar o estado nutricional e a gravidade da asma na primeira consulta, observou-se a forma grave apenas em pacientes com excesso de peso, enquanto Ventura et al. observaram a prevalência mais elevada de desnutrição no conjunto asma moderada/grave, de 1988 a 1993 no mesmo município, apesar da diferença dos critérios de classificação nutricional adotada na época.^28^

Em relação ao diagnóstico clínico de gravidade da doença na primeira consulta, a maior frequência foi de asma intermitente (51,5%) e grave em apenas 1,4%, o que demonstra uma proporção semelhante à citada na IV Diretrizes Brasileiras para o Manejo da Asma, 2006.^25^ Simões et al.,^29^ no município de Salvador, Bahia, descreveram uma prevalência mais elevada de asma leve (40%) e grave (10,8%) e mais baixa de intermitente (36%) e moderada (12,8%).

No presente estudo, foi avaliada a influência do IMC elevado e a gravidade da asma e, também, o controle da doença após um ano de acompanhamento dos pacientes no Programa. Feitas as análises, não foram demonstradas associações entre o estado nutricional e a gravidade da asma e entre o estado nutricional e o controle da doença.

Existe uma heterogeneidade nos estudos sobre a associação asma e obesidade devido ao uso de métodos diferentes para classificar a gravidade da asma, alguns usam sintomas referidos de sibilância, outros usam diagnóstico médico ou mensuração objetiva como espirometria e, portanto, demonstram resultados variados. Os autores Farah, Salome, em artigo de revisão, apontaram as controvérsias da associação obesidade e pior gravidade da asma.^11^ Cassol et al. realizaram um estudo populacional com adolescentes numa cidade da Região Sul do Brasil e não verificaram associação entre o aumento de IMC e piora dos sintomas da asma.^16^Nos EUA, Ross et al. não verificaram diferenças na função pulmonar entre crianças e adolescentes asmáticos obesos e não obesos.^17^ Por outro lado, outras pesquisas têm evidenciado associação entre IMC elevado e maior gravidade da asma.^18^^,^ 19

Em relação ao controle da asma, ainda não está bem estabelecida a associação entre a obesidade e pior controle da doença. Os autores Quinto et al. observaram que as crianças e adolescentes com sobrepeso e obesidade apresentaram pior controle da asma com mais exacerbações e maior uso de medicações de resgate.^21^ Kattan et al. descreveram em estudo com adolescentes asmáticos uma associação entre o pior controle da asma e a obesidade no sexo feminino.^20^Outros autores não observaram diferenças nos níveis de controle da asma entre os pacientes obesos e não obesos.^17^^,^ 30

O presente estudo avaliou a evolução da asma considerando os pacientes que compareceram a todas as consultas propostas, tendo em vista que as características desses pacientes possam ser diferentes daqueles que abandonaram o Programa e dos que faltaram às consultas, o que pode ter limitado as análises da evolução da doença. A quantificação dos números de crises pode ter sido influenciada pelo viés de memória, principalmente quando as exacerbações ocorreram várias vezes no período de um semestre; os atendimentos nas urgências constituem fatos marcantes na vida do paciente e tornam os registros mais fidedignos. Por outro lado, os pacientes iniciaram o seguimento no Programa em meses diferentes do ano, o que diminuiu o viés das exacerbações da asma pela sazonalidade.

Em um ano de acompanhamento 25,2% dos pacientes reduziram as crises e as procuras de atendimentos de urgência. Dos pacientes que apresentaram piora somente 19,1% tiveram mais crises e 17,6% necessitaram de mais atendimentos de urgências no período. Observou-se que a proporção de asmáticos que melhoraram foi maior dos que pioraram, o que aponta para o benefício do acompanhamento naqueles pacientes que aderiram ao Programa.

Ventura et al. verificaram que os asmáticos moderados e graves reduziram o número de crises ao analisar o período antes e após um ano de acompanhamento, nas mesmas UBS, de 1988 a 1993.^22^ Esses autores observaram, também, que o tempo de acompanhamento influenciou a evolução da asma moderada, o que demonstra uma melhoria do quadro clínico com tempo superior a um ano. Por outro lado, o tempo de acompanhamento menor ou maior do que um ano não mostrou diferença na evolução dos asmáticos graves.^22^ Os resultados encontrados concordaram com o estudo de coorte retrospectiva feito pelos autores Fontes et al., que avaliaram o acompanhamento das crianças e adolescentes asmáticos em serviço de pneumologia em Belo Horizonte, Minas Gerais, e observaram uma redução significativa na frequência de internações e atendimentos nas urgências por asma no período de um ano.^31^

De acordo com o presente estudo, a maioria dos pacientes apresentou adesão ao tratamento. Uma parcela importante desses resultados deve ser creditada ao fornecimento de medicamentos para asma pelas unidades de saúde de rede pública, incluindo o Município de Embu das Artes, desde 2009, com a Portaria n° 2.981 do Ministério da Saúde.^23^ Houve avanços nas políticas públicas de saúde com o reconhecimento dos gestores de saúde de a asma ser uma das doenças crônicas mais frequentes, disponibilizando para os pacientes asmáticos, medicamentos como corticoides inalatórios com ou sem associação com broncodilatadores de longa duração, medicamentos de resgate, como β-agonistas de curta duração e corticoides orais.

A melhoria da adesão dos pacientes e familiares ao Programa foi demonstrada pela redução da taxa de abandono de 53,2%^22^desde sua implantação nas duas UBS para atuais 17,8%. Essa constatação foi também descrita por Dalcin et al., em pacientes asmáticos recrutados do ambulatório de um hospital de Porto Alegre, Rio Grande do Sul. Observou-se, ainda, que o acesso ao medicamento e o uso adequado de corticoide inalatório associaram-se ao controle da asma.^30^

No entanto, apesar da consistente melhoria na adesão ao Programa e no acompanhamento, este estudo apresenta algumas limitações, como a obtenção de alguns dados secundários (prontuário), além da perda de informações sobre pacientes que faltaram ou abandonaram o Programa durante a coleta de dados do estudo. Apesar de tais limitações, pode-se concluir que não houve, no presente estudo, associações entre o estado nutricional, a gravidade e o controle da asma. A adesão ao tratamento possibilitou uma redução no número de crises de asma e nos atendimentos de urgência durante o período de acompanhamento, além da diminuição nas doses de corticoide inalatório. Este estudo ressalta a importância e a viabilidade do acompanhamento dos pacientes asmáticos em UBS. Além disso, a adoção de políticas públicas que facilitaram a introdução de medicamentos específicos para o tratamento da asma, doença crônica mais frequente durante a infância, na atenção básica em saúde contribui para os desfechos favoráveis dessa evolução.
